# LINC00467, Driven by Copy Number Amplification and DNA Demethylation, Is Associated with Oxidative Lipid Metabolism and Immune Infiltration in Breast Cancer

**DOI:** 10.1155/2021/4586319

**Published:** 2021-12-15

**Authors:** Hao Bo, Wancong Zhang, Xiaoping Zhong, Jiasheng Chen, Yang Liu, Kit-Leong Cheong, Pengju Fan, Shijie Tang

**Affiliations:** ^1^Department of Plastic and Esthetic Surgery, Xiangya Hospital of Central South University, Changsha, Hunan 410008, China; ^2^Key Laboratory of Reproductive and Stem Cell Engineering, National Health and Family Planning Commission, Institute of Reproductive and Stem Cell Engineering, Basic Medicine College, Central South University, Changsha, Hunan 410078, China; ^3^Clinical Research Center for Reproduction and Genetics in Hunan Province, Reproductive and Genetic Hospital of CITIC-Xiangya, Changsha, Hunan 410078, China; ^4^Department of Plastic Surgery and Burn Center, Second Affiliated Hospital, Shantou University Medical College, Shantou, Guangdong 515000, China; ^5^Plastic Surgery Institute of Shantou University Medical College, Guangdong 515000, China; ^6^Guangdong Provincial Key Laboratory of Marine Biotechnology, Department of Biology, College of Science, Shantou University, Shantou, Guangdong 515063, China

## Abstract

Breast cancer (BRCA) is a malignant tumor with a high incidence and poor prognosis in females. However, its pathogenesis remains unclear. In this study, based on bioinformatic analysis, we found that LINC00467 was highly expressed in BRCA and was associated with tumor metastasis and poor prognosis. The genomic and epigenetic analysis showed that LINC00467 may also be regulated by copy number amplification (CNA), chromatin openness, and DNA methylation. In vitro experiments showed that it could promote the proliferation, migration, and invasion of BRCA cells. Competitive endogenous RNA (ceRNA) regulatory network analysis and weighted gene coexpression network analysis (WGCNA) suggested that LINC00467 may play a role in signaling pathways of peroxisomal lipid metabolism, immunity, and others through microRNAs (miRNAs) targeting transforming growth factor beta 2 (TGFB2). In addition, copy number amplification and high expression of LINC00467 were associated with the low infiltration of CD8+ and CD4+ T cells. In conclusion, we found that LINC00467, driven by copy number amplification and DNA demethylation, may be a potential biomarker for the diagnosis and prognosis of BRCA and a tumor promoter acting as a potential therapeutic target for BRCA as well.

## 1. Introduction

Breast cancer (BRCA) is one of the most common malignancies affecting women worldwide, and approximately more than 1.3 million women will develop BRCA during their lifetime each year. BRCA also has a high mortality rate and continues to be the most common cause of death among female cancer patients in the world, causing 458,000 deaths every year [1], and its incidence continues to grow globally [2].

BRCA can be mainly divided into subtypes of luminal A, luminal B, normal breast-like, HER-2, and basal-like. The causes of BRCA are complicated, among which obesity and smoking are the most common risk factors [3, 4]. BRCA treatments include surgery, chemotherapy, hormonotherapy, radiotherapy, and immunotherapy; however, due to the intratumor heterogeneity in breast cancer, its pathogenesis has remained unclear. Furthermore, the predictive biomarker(s) for recurrence and metastasis of BRCA are still unavailable. Distal metastasis of the lung, liver, bone, and brain [5–7] from BRCA have been identified as the most common and troublesome consequences; therefore, it is of great significance to find new therapeutic targets and molecular biomarkers for BRCA.

LncRNA is a type of noncoding RNA (ncRNA) molecule with a length of more than 200 nucleotides. It can regulate the gene expression at the epigenetic, transcriptional, translational, and posttranslational levels. Studies have shown that lncRNA is dysexpressed and involved in the pathological process of a variety of cancers, including BRCA. LINC00152 acts as a tumor promoter in BRCA and other carcinomas [8], and LincROR has also been found to promote the invasion, migration, and drug resistance of BRCA through various signaling pathways such as EMT and MAPK/ERK [9–12]. In addition, LINC00673 can promote the proliferation of BRCA cells through the signaling pathway of miR-515-5p/MARK4/Hippo [13]. The above evidence suggests that lncRNA plays a crucial role in BRCA, but its mechanism remains unclear. Furthermore, few biomarkers of lncRNA related to BRCA are available in clinical practice.

In this study, through data mining of the public databases, including Gene Expression Omnibus (GEO), the Cancer Genome Atlas (TCGA), Molecular Taxonomy of Breast Cancer International Consortium (METABRIC), and Cancer Cell Line Encyclopedia (CCLE), we found that the LINC00467 expression rose significantly in BRCA and could be used as the potential diagnostic and prognostic biomarker for it. Furthermore, we confirmed LINC00467 as a tumor promoter in vitro and predicted the mechanism of LINC00467 dysexpression as well as its regulation of downstream signaling pathways based on bioinformatics analysis, providing a new insight for the development of biomarkers and therapeutic targets for BRCA.

## 2. Material and Methods

### 2.1. Meta-Analysis Based on the Databases of GEO and TCGA

Firstly, GSE7904 [14], GSE45827 [15], GSE65194 [16, 17], GSE22820 [18, 19], and GSE38959 were selected from the GEO database. Then, difference analysis was performed using GEO2R, with cut-off values of Log | FC | >1 and adjusted *p* value <0.05 for the screening of differential genes (Supplementary Table 1-5). Based on the Ensemble and RefSeq database annotation, the top 10 lncRNAs with significant differential expression were exacted, and heat maps were constructed through meta-analysis via the RobustRankAggreg of *R* package [20]. The TCGA-BRCA cohort in the Gene Expression Profiling Interactive Analysis (GEPIA) [21] database (http://gepia.cancer-pku.cn/index.html) was used to verify the 10 lncRNAs (5 upregulated and 5 downregulated, Supplementary Table 6). GSE57297 [22, 23], a dataset containing 25 BRCA samples and 7 nontumor samples, was downloaded from the GEO database, among which the data of 4 probes of LINC00467 were extracted (A_23_P1014, A_19_P00318494, A_33_P3223097, and A_19_P00318495), helping to further verify the differential expression of LINC00467 in BRCA. Similarly, GSE1299 [24] was downloaded from the GEO database, and the expression levels of LINC00467 in breast cancer cell lines were displayed. The expression levels of LINC00467 in BRCA cell lines (CCLE data) were download from the University of California Santa Cruz (UCSC) Xena [25] database (https://xena.ucsc.edu/).

### 2.2. Association Analysis between LINC00467 and BRCA

The ROC curves of the LINC00467 expression in TCGA-BRCA cohort obtained from the UCSC Xena database were calculated. GSE9893 [26] was downloaded from the GEO database to analyze the association between LINC00467 and BRCA metastasis. The LINC00467 expression in circulating tumor cells (CTCs), including GSE41245 [27] and GSE55807 [28], was downloaded from the ctcRbase [29] database (http://www.origin-gene.cn/database/ctcRbase/index). Kaplan–Meier survival curves of LINC00467 based on different datasets were present with Kaplan–Meier Plotter [30] (http://kmplot.com/analysis/index).

### 2.3. Data Mining of Single-Cell Sequencing (SCS)

GSE113197 [31, 32], a dataset of single-cell transcriptome sequencing of human breast, was downloaded from the Human Cell Landscape [33] database (http://bis.zju.edu.cn/ HCL/). SCS and its relevant enrichment analysis of BRCA CTCs were all performed by the CancerSEA [34] database (http://biocc.hrbmu.edu.cn/CancerSEA/home.jsp) according to the default parameters.

### 2.4. Analysis of Copy Number Variation (CNV) of LINC00467

The information data of patients, the expression data, and CNV data of LINC00467 in the TCGA-BRCA cohort and METABRIC cohort were downloaded from the cbioportal [35, 36] database (https://www.cbioportal.org/), as well as the expression data and CNV data of LINC00467 in the BRCA cell lines (CCLE data). Bar charts, pie charts, and box plots were displayed with ggplot2 [37] of the *R* package, while survival curves were calculated by survminer of the *R* package.

### 2.5. Epigenetic Mechanisms of LINC00467

Expression data of LINC00467, chromatin openness data, and methylation data of the promoter region in the TCGA-BRCA cohort were downloaded from the UCSC Xena database. H3K27ac, H3K4me3, and sensitivity data of DNA enzyme related to LINC00467 in the BRCA cell line MCF7 were downloaded and calculated with the UCSC Genome Browser [38] database (https://genome.ucsc.edu/index.html).

### 2.6. Cell Culture, siRNA Transfection, and 5-Aza-Deoxycytidine Treatment

The BRCA cell lines of MCF-7 and MDA-MB-231 were donated by the Cancer Research Institute of Central South University. Classically, Dulbecco's Modified Eagle's Medium (DMEM, GIBCO, NY - Grand Island, USA) containing 10% fetal bovine serum (FBS, GIBCO, NY - Grand Island, USA) and 1% penicillin-streptomycin is used as the culture medium in an incubator (5% CO_2_, 37°C). According to the protocol of Lipofectamine3000 (Lipo3K, Life Technologies, CA-Carlsbad, USA), short interfering ribonucleic acid (siRNA, RIBOBIO, Guangzhou, China) of LINC00467 and its negative controls (NC, RIBOBIO, Guangzhou, China) were transfected with a final siRNA concentration of 50 nM in a 6-well plate. Cells were collected 48 hours after transfection for subsequent experiments. When the cell density reached 70%, we treated the cells with 10 *μ*m concentration of 5-aza-deoxycytidine in a 6-well plate and collected the cell pellet after 48 hours.

### 2.7. RNA Isolation and Quantitative Real-Time PCR (qRT-PCR)

Cells were collected 48 hours after siRNA transfection and then washed with PBS. RNA was isolated by the TRIzol-based method (Life Technologies, CA-Carlsbad, USA). RNA integrity was verified by agar gel electrophoresis, while RNA concentration was detected using a nanopore spectrophotometer. Subsequently, the corresponding cDNA was synthesized using one microgram of qualified RNA per well as the template through a reverse transcription system kit. Then, qRT-PCR was performed using cDNA as the template through the Roche Transcriptor First Strand cDNA Synthesis Kit (Roche, Basel, Switzerland). Primer sequences were as follows: LINC00467 forward: 5′-TCGTCTTCAGGAAGCCAGAC-3′ and reverse: 5′-TGGAAATCAAAAGGGTCAGC-3′. *β*-Actin (ACTB) forward: 5′-CTGAGGATGCGAGGTTCTGCTTG-3′, reverse: 5′-GTCACCGGAGTCCATCACGAT-3′ (Sangon Biotech, Shanghai, China). *β*-Actin was used as the reference gene.

### 2.8. Cell Migration and Invasion by Transwell Assay

The ability to migrate and invade the capacities of cells was determined by transwell assay. For cell migration, 10,000 cells were inoculated above the 8 *μ*m chamber (Corning Inc., NY-Corning, USA) with a medium containing 200 microliters of 1% FBS and below the chamber with a medium containing 15% FBS of 800 microliters for 24 hours. For cell invasion, different from cell migration, the number of inoculated cells was increased up to 20,000, 1 : 5 diluted matrix glue was added above the chamber, and cells were incubated for 36 hours under the same medium with cell migration. Finally, five fields were randomly selected to take photos, and the number of cells passing through the chamber was counted.

### 2.9. WGCNA and Pathway Enrichment Analysis

Compared with traditional coexpression analysis, WGCNA is more suitable for constructing gene coexpression network in large samples and can construct more stable coexpression network [39]. So, it has great advantages in finding phenotype-related gene modules. In this study, gene expression data of the TCGA-BRCA cohort were downloaded from the UCSC Xena database, and the genes with the top 75% of the median absolute deviation were selected for subsequent WGCNA analysis. The WGCNA of the *R* package was used to construct the network with a power of 3. All the genes in the module where LINC00467 was located were extracted. Pathway enrichment analysis was carried out using Metascape software (http://metascape.org/gp/index.html#/main/step1) in accordance with the default parameters [40].

### 2.10. Analysis of Competitive Endogenous RNA (ceRNA) Network Driven by the CNV of LINC00467

Data analysis of ceRNA regulation network, enrichment, and ceRNA prognosis of LINC00467 was all performed by LnCeVar (http://www.bio-bigdata.net/LnCeVar/index.jsp) based on the TCGA-BRCA cohort according to the default parameters [41]. The expression correlation data of LINC00467 and TGFB2 (METABRIC cohort) were downloaded from the cbioportal database. Symbiosis of the copy number of both LINC00467 and TGFB2 was analyzed by cBioportal.

### 2.11. Analysis of Immune Cell Infiltration

Analysis of immune cell infiltration associated with different types of CNV of LINC00467 was performed by the TIMER2.0 [42] database (http://timer.cistrome.org/), while analysis of immune cell infiltration associated with the expression levels of LINC00467 was performed by the ImmLnc [43] database (http://bio-bigdata.hrbmu.edu.cn/ImmLnc/index.jsp). BRCA is a very common type of tumor. These two tools, TIMER2.0 and ImmLnc, are also developed for analysis of immune cell infiltration in common tumor types including BRCA. Thus, both were performed based on TCGA-BRCA cohort according to the default parameters.

### 2.12. Statistical Analysis

The Student *t*-test was used for comparison between two groups, and ANOVA was used for comparison among groups. The descriptive data were expressed as mean ± standard error (SE). Statistical analysis and graphs were performed by GraphPad Prism, *R* statistical software, or bioinformatics online tool (http://www.bioinformatics.com.cn/). *p* value <0.05 was considered significantly different for *t*-test and ANOVA. Adjusted *p* value <0.05 was considered significantly different for multiple tests.

## 3. Results

### 3.1. The Significant Upregulation of the LINC00467 Expression in BRCA

**Figure 1:**
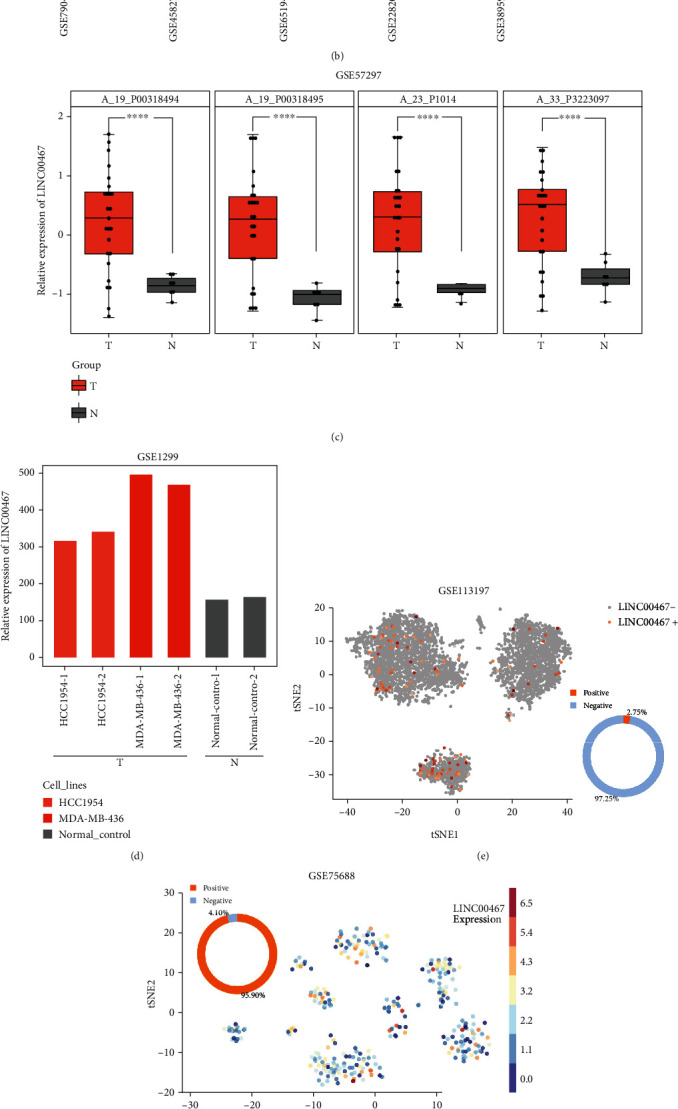
Upregulation of the LINC00467 expression in BRCA based on the integration of gene chips and RNA sequencing. (a) Flow chart: after the five gene chips of GEO were, respectively, analyzed with GEO2R, differentially expressed lncRNAs were screened out based on annotations of the Ensemble and RefSeq database, and then the RRA of the *R* package was used to screen the significantly different molecules in multiple datasets. (b) The top 10 differentially expressed lncRNAs based on RRA analysis (five upregulated and five downregulated) were calculated in the heat maps. (c) Another dataset of GEO was used to verify the differential expression of LINC00467 (samples of tissues). (d) Gene chips of GEO were used to verify differential expression of LINC00467 (samples of cell lines). (e) LINC00467 expression in breast cells (extreme low positive rate). (f) LINC00467 expression in BRCA (extreme high positive rate). (g) LINC00467 expression in BRCA CTCs. (h) Correlation between LINC00467 and phenotypes of BRCA CTCs based on the analysis of GSE75367. ^∗∗∗∗^*p* < 0.0001.

Meta-analysis based on GEO was conducted to find the 10 lncRNAs with the most significant difference and the most stable expression in 5 groups of gene chips (Figure 1(a)). We selected five upregulated and five downregulated lncRNAs, including LINC00467 and MALAT1 (Figure 1(b)), as candidates for further study. Expression data in BRCA from the GEPIA database showed that all of the lncRNAs were significantly different expressed except for MAIT; however, the trend of the differential expression of MALAT1 was not consistent with the gene chips of GEO (Supplementary Figure. 1A, 1B). Based on the gene chips of the GEO and GEPIA databases, we recognized LINC00467 as the molecule whose expression was upregulated most significantly and selected it for further study. Furthermore, we also verified the differential expression of LINC00467 in another GEO chip GSE57297 (the results of four probes of LINC00467 were consistent, as shown in Figure 1(c)). Compared with nontumor samples, the LINC00467 expression was also significantly upregulated in BRCA cell lines of HCC1954 and MDA-MB-436 (Figure 1(d)). We also found that the expression of LINC00467 in normal breast epithelial cell lines was lower than that of breast cancer cell lines based on CCLE data (Supplementary Figure. 1C). These results suggested that LINC00467 may be a good biomarker for BRCA.

Single-cell sequencing (SCS) data showed that there were multiple cell types in both breast and BRCA. In order to explore the cell types where LINC00467 was expressed, we analyzed the data of SCS of breast tissues in the Human Cell Landscape database and that of BRCA tissues in the CancerSEA database, respectively, showing that the positive rate of LINC00467 in normal breast cells was very low, only 2.75% (Figure 1(e)), while the positive rate of LINC00467 in BRCA cells was high, reaching up to 95.9% (Figure 1(f)). What is more, the positive rate of LINC467 in circulating tumor cells (CTCs) of BRCA was 78.57% (Figure 1(g)), suggesting that LINC00467 may be related to the occurrence and metastasis of BRCA. In addition, we also analyzed the relationship between LINC00467 and tumor-related signaling pathway using CancerSEA, finding that the LINC00467 expression was significantly negatively correlated with the differentiation, rest, inflammation, and apoptosis of BRCA CTCs (Figure 1(h)), suggesting that LINC00467 may also play a role in promoting the survival of CTCs.

### 3.2. Biomarker Value of LINC00467 in BRCA

**Figure 2:**
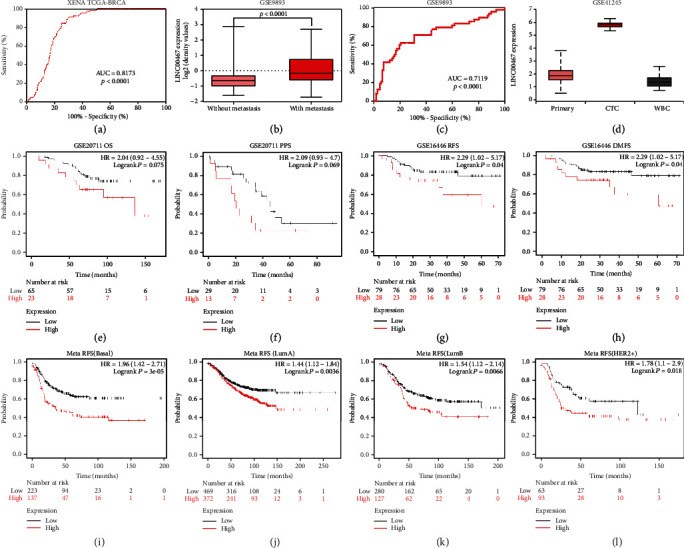
Diagnostic and prognostic values of LINC00467 in BRCA. (a) The diagnostic value of LINC00467 for BRCA based on TCGA BRCA cohort in the XENA database. (b) High expression of LINC00467 in metastatic BRCA based on GSE9893. (c) LINC00467 expression could distinguish metastatic and nonmetastatic tumor tissues. (d) LINC00467 expression was significantly higher in CTCs than in BRCA in situ. (e)–(h) The correlation between LINC00467 expression and OS/PPS/RFS/DMFS in different BRCA datasets. (i)–(l) The correlation between LINC00467 expression in different types of samples and RFS in multiple datasets.

In order to explore the clinical value of LINC00467, we analyzed the diagnostic specificity and sensitivity of LINC00467 for BRCA based on the UCSC Xena database, finding that the LINC00467 expression could distinguish tumor and non-tumor tissues very well (Figure 2(a)). Besides, data analysis of GEO found that LINC00467 was significantly more highly expressed in metastatic BRCA tissues (Figure 2(b)), with good diagnostic specificity and sensitivity (Figure 2(c)). In addition, we also found that LINC00467 was significantly overexpressed in CTCs of BRCA (Figure 2(d), Supplementary Figure 2). These results suggested that LINC00467 may be an excellent biomarker for the diagnosis and metastasis of BRCA.

Furthermore, we analyzed the association between LINC00467 and BRCA prognosis with Kaplan–Meier Plotter. Based on the analysis of the independent dataset, we found that LINC00467 was correlated with four prognostic parameters of BRCA (OS:overall survival/PPS: postprogression survival/RFS: relapse-free survival/DMFS: distant metatasis-free survival). The higher the expression of LINC00467, the lower the four parameters of BRCA would be (Figures 2(e)–2(h)). In addition, a meta-analysis of multiple groups of RFS data in Kaplan–Meier Plotter revealed that LINC00467 had a prognostic value for RFS among different pathological types of BRCA (Basal/LumA/LumB/Her2+) (Figures 2(i)–2(l)).

### 3.3. Genomic Copy Number Amplification of LINC00467 in BRCA

**Figure 3:**
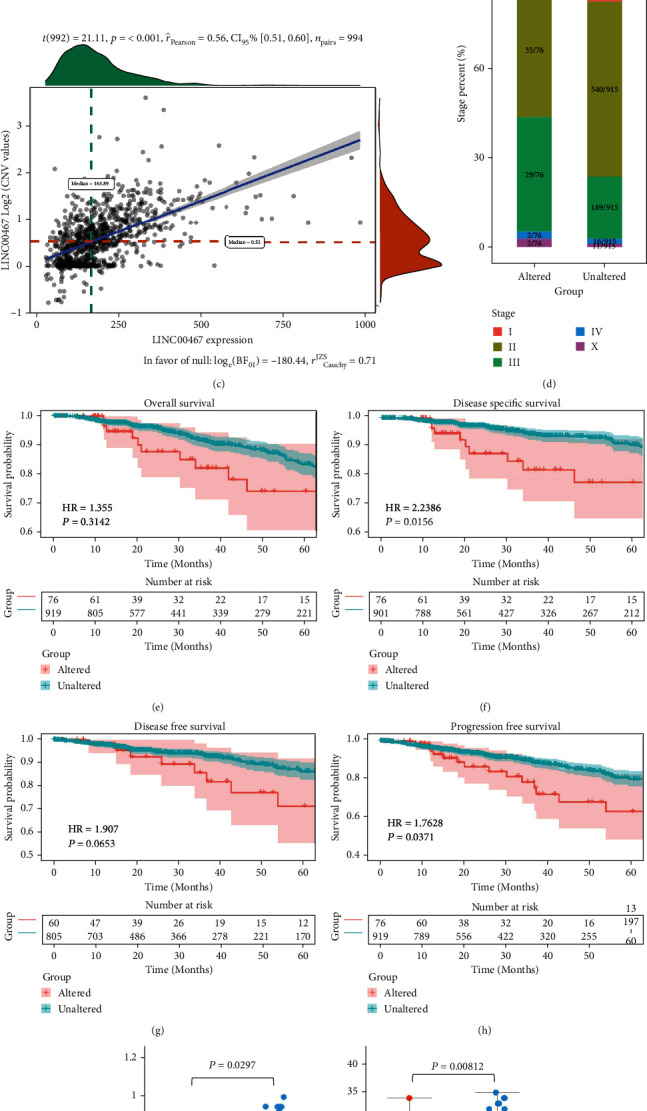
Analysis of LINC00467 CNV in BRCA. (a) LINC00467 has different frequencies of copy number amplification in different types of BRCA. (b) Relationship between LINC00467 expression and types of CNV. (c) The positive correlation between LINC00467 expression and its copy number level. (d) The positive correlation between copy number amplification of LINC00467 and stages of diseases. (e)–(h) Patients with LINC00467 amplified had a worse prognosis. (i) Patients with LINC00467 amplified showed more genomic changes. (j) Patients with LINC00467 amplified had higher aneuploidy scores. (k) LINC00467 showed a higher frequency of amplification in BRCA cell lines. (l) LINC00467 was more highly expressed in the amplified cell lines.

Studies have proven that a lot of lncRNAs had dose effects and were regulated by the genomic CNV [44, 45]. Therefore, we analyzed BRCA data in the Pan-Cancer Atlas based on TCGA and found that LINC00467 had a certain frequency of amplification in different types of BRCA, with the highest frequency in invasive lobular carcinoma, up to approximately 15% (Figure 3(a)). Furtherly, we analyzed the LINC00467 expression in different types of genomic variation, finding it was significantly higher in the increased or amplified genome than that in the deleted or normal diploid samples (Figure 3(b)), and significantly positively correlated with its CNV value (Figure 3(c)). In addition, we analyzed the correlation between the CNV of LINC00467 and the clinicopathological characteristics of BRCA, finding that patients with CNV of LINC00467 were more likely to be at advanced Stage IV and Stage X of the tumor (Figure 3(d)). CNV of LINC00467 was also associated with the prognosis of the patients with BRCA, including OS, disease-specific survival (DSS), disease-free survival (DFS), progression-free survival (PFS), and patients with copy number amplification of LINC00467 that had a worse prognosis (Figures 3(e)–3(h)). In addition, we found that patients with copy number amplification of LINC00467 had greater genomic fragment changes and aneuploidy scores (Figures 3(i) and 3(j)), suggesting that LINC00467 may be associated with genomic instability of BRCA. Similarly, in the BRCA cell lines (based on CCLE data), LINC00467 also showed a high frequency of amplification, reaching up to 15.69% (Figure 3(k)), and was significantly more highly expressed in the samples with copy number amplification (Figure 3(l)). To verify the above results, we also analyzed the CNV of LINC00467 in another dataset of breast cancer (TCGA Firehose Legacy) finding that LINC00467 had a high frequency of copy number amplification in BRCA (Supplementary Figure. 3A), and the patients with dose-dependent effect and copy number amplification had a worse prognosis (Supplementary Figure. 3B-3D). Patients with copy number amplification of LINC00467 also had a higher proportion of advanced stages and more changes of genome fragments (Supplementary Figure. 3E-3G). These results fully indicated that genome amplification of LINC00467 may be one of the causes of upregulation of LINC00467 expression, LINC00467 may be one of the driver genes of BRCA, and its copy number amplification may be a molecular biomarker of metastasis and recurrence for BRCA patients as well.

### 3.4. Epigenetic Regulation of LINC00467

**Figure 4:**
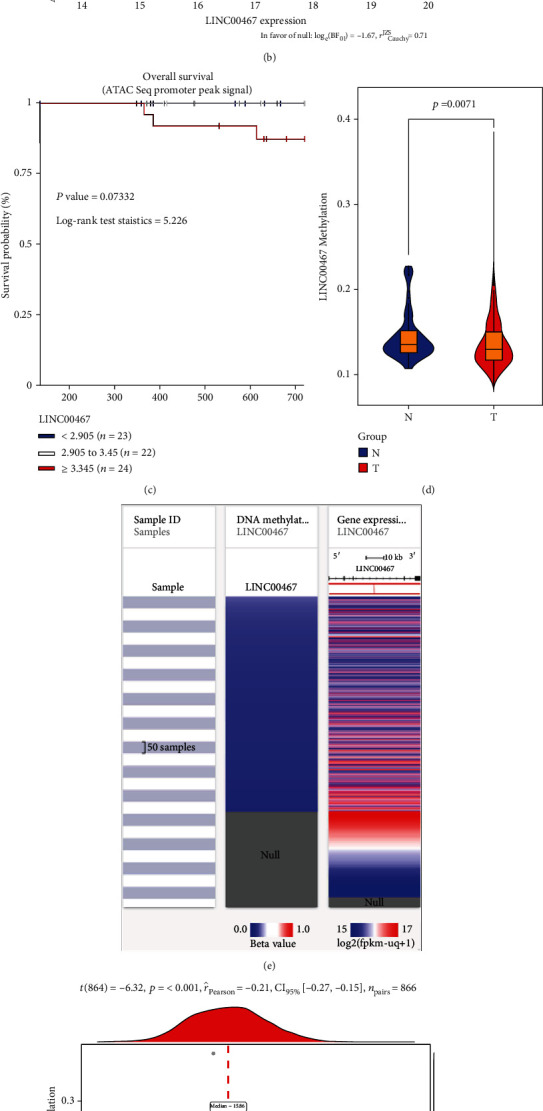
Epigenetics and transcriptional regulatory mechanisms of LINC00467. (a) Heat map of the correlation between chromatin openness and LINC00467 expression based on the BRCA ATAC-Seq data and the expression data of the UCSC Xena database. (b) Correlation analysis of chromatin openness and expression level in LINC00467 promoter. (c) Association of chromatin openness of LINC00467 and the prognosis. (d) The significant differences of the methylation in LINC00467 promoter of BRCA. (e) Heat map of correlation between the methylation in LINC00467 promoter and LINC00467 expression. (f) The significant correlation between the methylation in the promoter and the expression of LINC00467. (g) The expression of LINC00467 in 5-aza-deoxycytidine treated cells was detected by qRT-PCR. (h) Active histone modifications of H3K27ac and H3K4me3 and sensitivities of DNA enzyme near the LINC00467 promoter in BRCA cells based on UCSC. ^∗^*p* < 0.05.

Studies have shown that the expression of lncRNA is regulated by epigenetic factors such as DNA methylation and histone modification [46, 47]. Therefore, we analyzed the relationship between LINC00467 and its epigenetic modifications based on the TCGA BRCA cohort, finding that samples with high expression of LINC00467 also had high chromatin openness in the promoter (Figure 4(a)), and there was a close correlation between the LINC00467 expression and its chromatin openness (Figure 4(b)). Besides, patients with the high expression of LINC00467 had a lower 5-year overall survival rate (Figure 4(c)). These results suggest that epigenetic regulation may indeed be associated with a high expression of LINC00467. Furtherly, we measured the average methylation level of the promoter of LINC00467, finding it was lower in tumor samples (Figure 4(d)) and significantly negatively correlated with the LINC00467 expression (Figures 4(e) and 4(f)). After MCF-7 cells treated with the DNA methyltransferase inhibitor 5-aza-deoxycytidine, we detected the expression of LINC00467 and found that the expression of LINC00467 was significantly upregulated (Figure 4(g)). In addition, we also found significant enrichment of H3K27ac/H3K4me3 (activated epigenetic modification) and peaks in the sensitivity of DNA enzyme near the LINC00467 promoter (Figure 4(h)). These results suggest that epigenetic modifications, including methylation and histone modifications, may lead to high expression of LINC00467.

### 3.5. Proliferation, Migration, and Invasion of BRCA Cells Inhibited by Silencing LINC00467

**Figure 5:**
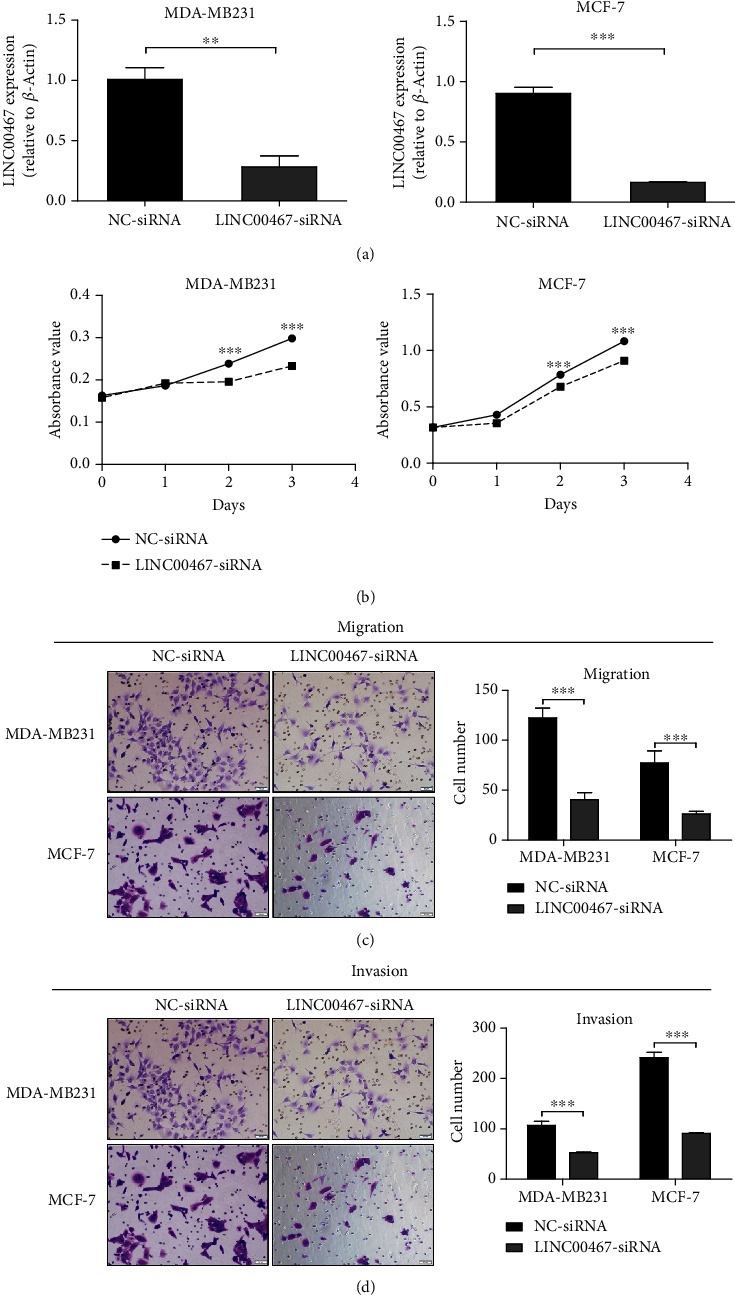
Silencing LINC00467 inhibits the malignant phenotypes of BRCA. (a) qRT-PCR was used to detect the silencing effect of LINC00467. (b) Changes in cell proliferation were detected by MTT assay after LINC00467 silenced. (c) Changes in cell migration were detected by transwell assay after LINC00467 silenced. (d) Changes in cell invasion were detected by transwell assay after LINC00467 silenced. ^∗∗^*p* < 0.01, ^∗∗∗^*p* < 0.001.

In order to investigate the biological functions of LINC00467 in BRCA, we conducted the tests of cell migration and invasion using transwell assay after LINC00467 was silenced in BRCA cell lines (Figure 5(a)), and finding the proliferation ability of BRCA cells was significantly reduced after LINC00467 was silenced (Figure 5(b)). Similarly, we found that the number of migrating cells in LINC00467 siRNAs was significantly fewer than that in its NC group (Figure 5(c)), and their invasion ability was also significantly reduced (Figure 5(d)). These results confirmed that LINC00467 could promote the migration and invasion of BRCA cells.

### 3.6. Screening of the Downstream Signals Regulated by LINC00467

**Figure 6:**
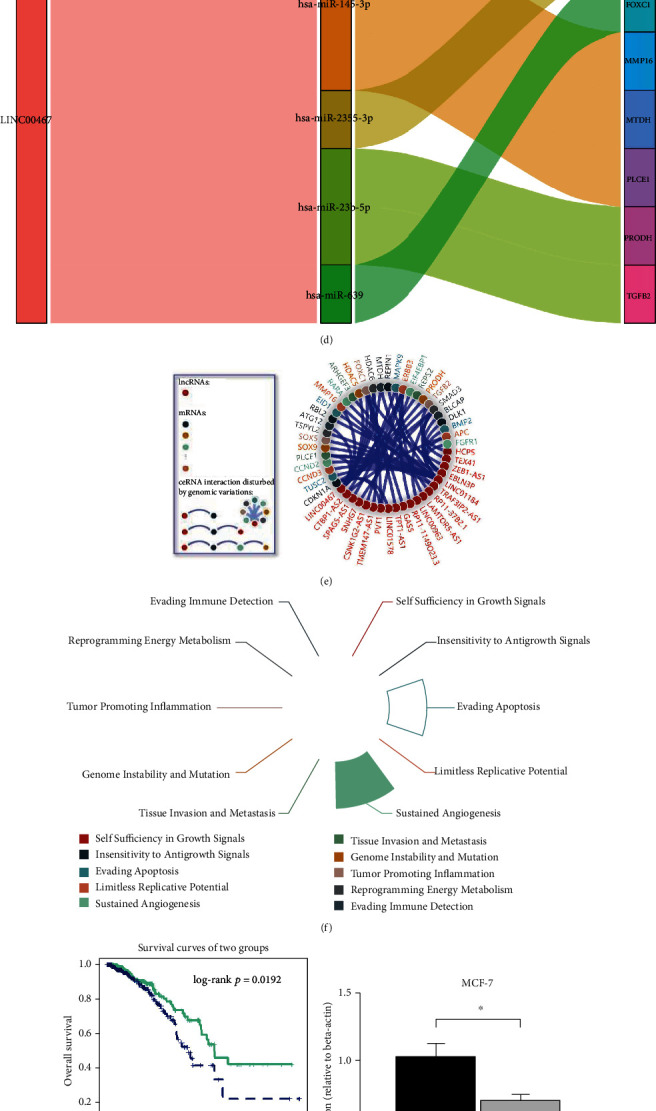
Biological processes, signaling pathways, and ceRNA regulatory network associated with LINC00467. (a) Hierarchical clustering and module clustering based on gene expression. (b, c) GO and KEGG Enrichment analysis of the genes at the module where LINC00467 is located. (d) CeRNA regulatory network of LINC00467 in BRCA using LnCeVar and the Sankey diagrams presenting the correlated primary network. (e) The secondary network of ceRNA mediated by LINC00467. (f) enrichment analysis of immunity, apoptosis escape, and other signaling pathways in ceRNA network. (g) Patients with high expressions of both LINC00467 and TGFB2 had a lower overall survival rate. (h) The location of LINC00467 and TGFB2 on chromosomes, based on UCSC. (i) Frequent co-amplification of LINC00467 and TGFB2 based on TCGA BRCA cohort. ^∗^*p* < 0.05.

In order to explore the downstream signals regulated by LINC00467, we downloaded the data of the TCGA BRCA cohort and performed WGCNA. First, we filtered with the values of power and recognized 3 was the most appropriate power value (Supplementary Figure. 4A). Then, based on gene coexpression analysis, a total of 25 coexpression modules were obtained after merging their cluster trees (Figure 6(a)), with LINC00467 in the red module. Based on coexpressed molecules often coparticipate in some biological processes or pathways, we carried out tissue-specific and cell-specific enrichment analysis. We subsequently showed that this red module's genes were mainly concentrated in breast cells and BRCA cells MCF-7 (Supplementary Figure. 4B), which was consistent with the high positive rate of LINC00467 in BRCA cells previously found in the SCS. Enrichment analysis revealed that these specific genes were mainly regulated by TFs such as FOXA1/TP53/TWIST1, which were highly related to the occurrence or progression of tumors (Supplementary Figure. 4C). The biological functions of these genes were mainly served for the phenotypes related to the development, proliferation, and growth of cells (Figure 6(b)). Finally, KEGG enrichment analysis showed that these molecules were mainly concentrated in signaling pathways related to tumorigenesis, lipid peroxide metabolism, or immunity, such as peroxisomal lipid metabolism, metabolism of lipids, antigen presentation, P53, and NOTCH (Figure 6(c)).

Previous studies have shown that LINC00467 may be involved in the regulation of gene expression through competing endogenous RNA (ceRNA) [48, 49]. Therefore, we speculated that LINC00467 may also play a role in BRCA through ceRNA. Based on the TCGA BRCA cohort, we analyzed ceRNA regulatory network mediated by LINC00467 using LnCeVar online tool and found that LINC00467 could regulate the gene expression through multiple miRNAs (Figure 6(d)), and a more multilevel regulatory network was further formed (Figure 6(e)), exerting a profound and lasting influence. Subsequently, enrichment analysis showed that the LINC00467-mediated ceRNA regulatory network functions were mainly concentrated in the malignant biological phenotypes of growth, apoptosis escape, migration/invasion, immune escape, and genomic instability, which were closely related to the tumors (Figure 6(f)). Among these ceRNAs, there was an important molecule, TGFB2, existing in the TGF-*β* signaling pathway. And the expression correlation between LINC00467 and TGFB2 was also been further validated in the METABRIC data (Supplementary Figure. 4D). BRCA patients with the high TGFB2 expression showed a significantly lower overall survival rate (Figure 6(g)). Additionally, it is interesting that both TGFB2 and LINC00467 were located on the short arm of chromosome 1 (Figure 6(h)), and they were co-amplified in BRCA samples (Figure 6(i)).

### 3.7. Immune Regulation of BRCA by LINC00467

**Figure 7:**
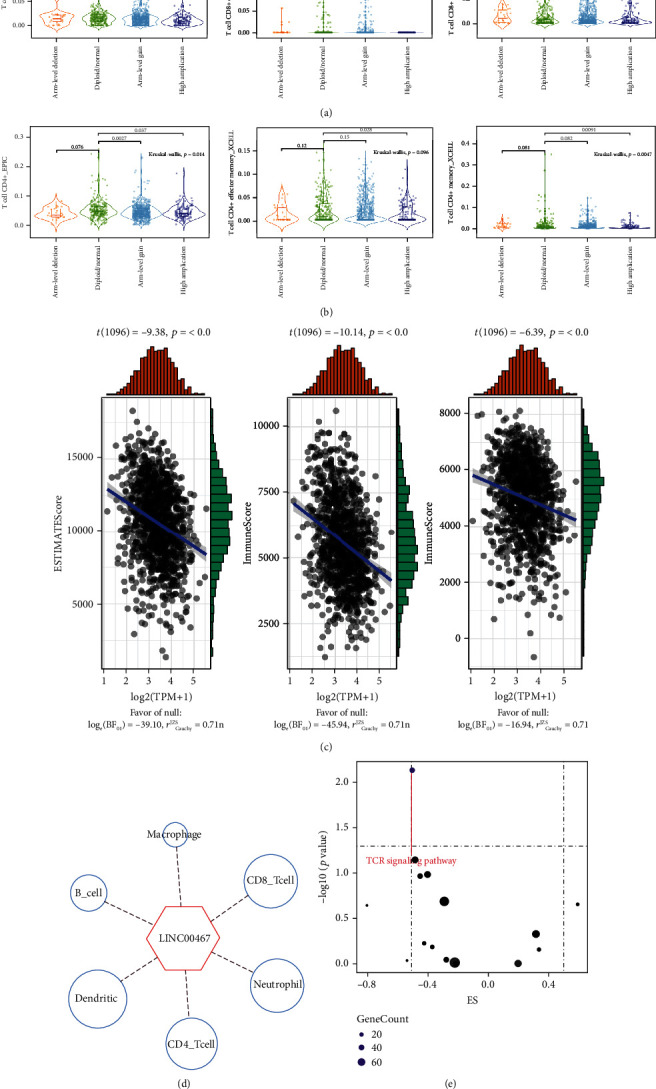
Genome amplification and expression levels of LINC00467 were associated with T cell infiltration. (a) CD8+ T cells showed lower infiltration in samples with LINC00467 amplified based on the TIMER2.0 database. (b) CD4+ T cells showed lower infiltration in samples with LINC00467 amplified based on the TIMER2.0 database. (c) The LINC00467 expression was significantly negatively correlated with ESTIMATE Score/Immune Score/Stromal Score based on the TIMER2.0 database. (d) The LINC00467 expression in the TCGA BRCA cohort was negatively correlated with a variety of immune cells based on the ImmLnc database. (e) GSEA enrichment analysis based on the ImmLnc database showed that the LINC00467 expression in TCGA BRCA cohort had the highest negative correlation with the TCR signal pathway.

Studies have shown that many lncRNAs were involved in immune regulation of the tumors [50, 51]. However, whether LINC00467 was involved in tumor immunity of BRCA has remained unknown. Therefore, we analyzed the correlation between LINC00467 and the immune cells within the tumor microenvironment (TME) based on the TCGA BRCA cohort. Grouping according to the CNV types of LINC00467, we found that infiltrations of CD8+ T cells, CD8+ effector memory T cells, and CD8+ central memory T cells were all significantly reduced in the highly amplificated LINC00467 group (Figure 7(a)). Meanwhile, infiltrations of CD4+ T cells, CD4+ effector memory T cells, and CD4+ central memory T cells were also significantly reduced (Figure 7(b)). These results suggested that the genomic variation of LINC00467 may be related to the infiltration of immune cells. Our previous analysis found that LINC00467 was a dose-dependent gene. Further analysis found that LINC00467 was significantly negatively correlated with the immune scores and stromal scores of BRCA samples (Figure 7(c)). At the same time, we also found that LINC00467 was significantly negatively correlated with infiltrations of various immune cells, such as CD8+ T cells, CD4+ T cells, and macrophages (Figure 7(d)) and significantly negatively correlated with the TCR signaling pathway (Figure 7(e)). Based on these results, we speculated that LINC00467 might be a negative regulator of anti-tumor immunity, which promoted tumor progression by inhibiting the infiltration of immune cells.

## 4. Discussion

Global Cancer Statistics 2018 show that the incidence of breast cancer was 46.3%, and the mortality rate was 13.0%, remaining the top cause of death among female cancer patients [52]. Patients of breast cancer mainly die of metastasis of BRCA. However, we still lack effective biomarkers to predict the metastasis [53]. In our study, by integrating BRCA data from multiple databases, we found for the first time that LINC00467 was significantly more highly expressed in BRCA. Further analysis showed that LINC00467 was mainly expressed in BRCA cells and its CTCs, but not in normal tissues. The expression level of LINC00467 can effectively distinguish whether the tumor has metastasized or not. In addition, LINC00467 was significantly more highly expressed in CTCs of BRCA, and the survival rate of patients with high expression of LINC00467 was significantly lower than that of patients with low expression of LINC00467. These results indicated for the first time that LINC00467 was a promising biomarker predicting BRCA. One of our published studies and other studies also found LINC00467 to be the biomarker for the metastasis and recurrence of lung cancer [47], colorectal cancer [54], and glioma [55]. In conclusion, LINC00467 could be a molecular biomarker of the metastasis and recurrence of multiple tumors, needing to be further studied.

Studies have shown that the genome of breast cancer tissue was highly unstable and had many types of variation. Data from multiple cohorts showed high-frequency amplification in 1q32 [56–58], suggesting that this region may be the location of tumor driver genes. Coincidentally, LINC00467 was also located in this region. Analysis of BRCA data from the cBioPortal database found that there were different levels of LINC00467 amplification in breast cancer, presenting a dose-dependent effect with the LINC00467 expression. These results proved that the LINC00467 expression was driven by its CNV, and patients with copy number amplification of LINC00467 had a poor prognosis, which fully indicated that LINC00467 may be a driver gene of breast cancer. An indepth study of the carcinogenic mechanism of LINC00467 would provide new ideas and targets for the treatment of BRCA.

Epigenetic regulation was involved in the development and progression of a variety of tumors, as well as the expression of a variety of RNAs, including lncRNA. Corces et al. constructed the open chromatin atlas of various tumors and described their uniqueness using ATAC-Seq, revealing the heterogeneity of the gene expression between different cancers to some extent [59]. We downloaded the BRCA data from the UCSC Xena database and found that the LINC00467 expression was significantly positively correlated with the level of chromatin openness in the promoter, suggesting that LINC00467 may be regulated by epigenetic modifications, among which DNA methylation was the most common and apparent one. A number of studies have confirmed that DNA methylation played an important role in the expression of lncRNA [46, 60]. We found that the expression of LINC00467 was significantly negatively correlated with the average methylation level of the promoter, indicating that DNA methylation of LINC00467 may also be one of the mechanisms for its up-regulation, which was the second new discovery of our team, considering that the LINC00467 expression could be regulated by histone modification as the first discovery in our previous study, fully demonstrating the complexity of regulation of the LINC00467 expression. Consistent with the previous results, we also found the presence of active histone modifications in LINC00467 promoters in this study, such as H3K27ac/H3K4me3. In conclusion, we found for the first time that LINC00467 may be a molecule regulated by multiple factors, which provided a new perspective for the study of the regulation mechanism of lncRNA expression.

Similar to the findings in lung cancer, glioma, liver cancer, and other cancers, our experiment showed that LINC00467 could also significantly promote the migration, invasion, and proliferation of BRCA in vitro. In addition, LINC00467 has been shown to promote chemotherapy resistance in colon and liver cancer, suggesting that the function of LINC00467 may be slightly different in different cancers, but in general, it acts in a similar manner as a tumor promotion gene, suggesting that LINC00467 may be a therapeutic target for multiple tumors.

The results of WGCNA showed that LINC00467 may affect the biological processes, such as cell proliferation and growth of BRCA, and could also participate in signal pathways related to cell proliferation and cell cycle transformation, such as TP53 and Notch, which was similar to the result of the research about LINC00467 regulating TP53 to promote glioma progression [61]. In addition, we also found that LINC00467 may also be involved in the regulation of lipid peroxide metabolism and epithelial-mesenchymal transition and its relevant signaling pathways. This suggests that LINC00467 may affect the metabolic reprogramming of BRCA cells, thereby improving the antioxidant capacity of cells and promoting cell proliferation. CeRNA hypothesis was one of the common molecular mechanisms of lncRNAs, through which a number of lncRNAs located in the cytoplasm could adsorb miRNAs, so as to regulate the gene expression [62, 63]. Zheng et al. found that LINC00467/miR-18a-5p/NEDD9 could promote the malignant growth and migration of HCC [64]. In head and neck squamous cell carcinoma, some researchers have also found that LINC00467 could promote EMT and malignant progression of tumors through ceRNA hypothesis [65], indicating that LINC00467 may be able to combine with different miRNAs, and ceRNA hypothesis may be one of the important functional mechanisms for LINC00467. Similarly, based on LncCeVar database, we first identified the LINC00467-miR-23b-5p-TGFB2 axis as a link of LINC00467 to the occurrence of BRCA. For the patients with high expression of this axis, they had a poor prognosis, and interestingly, LINC00467 was co-amplified with TGFB2, suggesting that the amplification of both LINC00467 and TGFB2 in this axis and their stable expression may be one of the important reasons for BRCA metastasis and recurrence. The treatment targeting this axis may be effective for the metastasis and recurrence of breast cancer. At the same time, our findings also provide new evidence for the regulation mechanism of ceRNA by LINC00467, which organically links genome-level changes with epigenetic regulation.

Tumor immunotherapy has become a common treatment for refractory and recurrent BRCA, but the responses to immunotherapy vary greatly among different patients. Multiple studies have shown that lncRNA could participate in the regulation of immune cells of TME by regulating the survival of T cells and the process of antigen presentation [50, 51]. Our results showed that both copy number level and expression level of LINC00467 were significantly negatively correlated with the infiltration of CD4+ and CD8+ T cells. Furthermore, LINC00467 was significantly negatively correlated with the TCR signaling pathway. These results suggested that LINC00467 was likely to inhibit antitumor immunity by inhibiting the immune infiltration of T cells and the TCR signaling pathway. The amplification and high expression of LINC00467 may also be biomarkers of both the infiltration of T cells and the response of immunotherapy.

## 5. Conclusions

**Figure 8:**
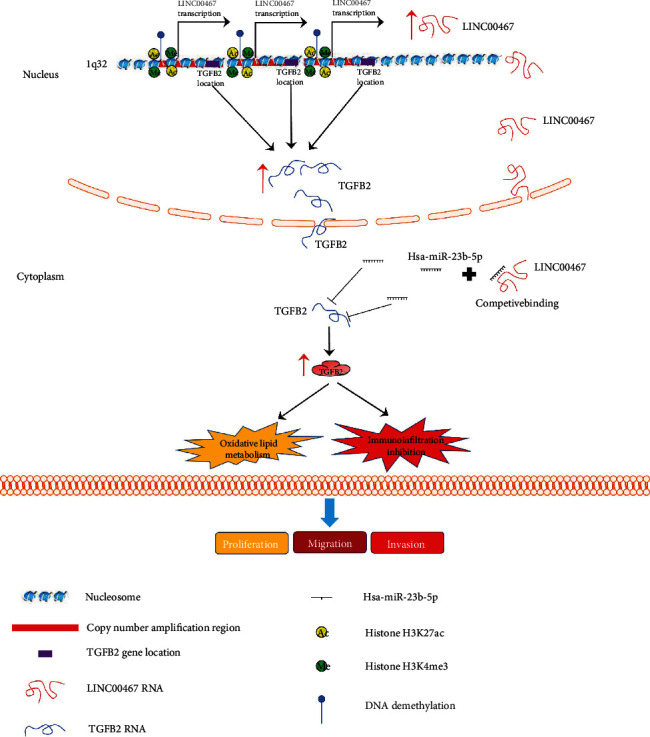
The potential mechanism of LINC00467 regulation in BRCA cells.

In conclusion, based on bioinformatic analysis, a molecular biomarker, LINC00467, was screened out with the values of diagnosis, metastasis, and recurrence for BRCA. In addition, it was also found that LINC00467 may be a tumor driver gene in BRCA and may be involved in the regulation of tumor immunity and lipid peroxide metabolism through ceRNA hypothesis (Figure 8). Treatment targeting LINC00467 and its downstream signaling pathways may be a new direction for the research and development of drugs for BRCA.

## Data Availability

Some of the data and figures (Figures 1(e)-1(h), Figure 2, Figures 3(i)-3(k), Figures 4(a), 4(c), 4(e), and 4(h), Figures 6(b), 6(c), 6(e)-(6g), and 6(i), Figures 7(a)-7(d), supplementary figure 1, supplementary figure 2, supplementary figure 3F/G, supplementary figure 4B/C) used to support the findings of this study are generated and downloaded from online tools. We have provided the Web links of these online tools in the material methods section of the main text. All related scripts and supported data are available for download at https://github.com/Bohao1990/4586319.

## Supplementary Materials

Supplementary Materials Supplementary Figure 1: Validation of differentially expressed lncRNAs in GEPIA BRCA dataset. (a, b) Differential expression was verified with GEPIA, with LINC00467 showing the most significant difference. (c) Expression of LINC00467 in BRCA cell line and normal mammary epithelial cell line of CCLE database. N: normal; T: tumor. Supplementary Figure 2: Expression of LINC00467 in CTCs of BRCA. LINC00467 expression in CTCs and BRCA in situ based on GSE41245. Supplementary Figure 3: TCGA Firehose Legacy dataset verifies the finding that LINC00467 had a high frequency of copy number amplification in BRCA. (a) LINC00467 has different frequencies of copy number amplification in different types of BRCA in Firehose Legacy dataset. (b) Relationship between LINC00467 expression and types of CNV. (c) The positive correlation between LINC00467 expression and its copy number level. (d) Patients with LINC00467 amplified had a worse overall survival. (e) The positive correlation between copy number amplification of LINC00467 and grades of BRCA. (f) Patients with LINC00467 amplified showed bigger lymph node examined number. (g) Patients with LINC00467 amplified showed more genomic changes. Supplementary Figure 4: WGCNA, enrichment, and correlation analysis. (a) Screening of the best soft thresholding with WGCNA. (b) Cell-specific and tissue-specific enrichment analysis of the genes at the red module where LINC00467 is located. (c) TF prediction of genes within the regulatory module. (d) Correlation analysis of LINC00467 and TGFB2 expression. Supplementary Table 1: Differentially expressed genes of GSE7904 dataset. Supplementary Table 2: Differentially expressed genes of GSE22820 dataset. Supplementary Table 3: Differentially expressed genes of GSE38959 dataset. Supplementary Table 4: Differentially expressed genes of GSE45827 dataset. Supplementary Table 5: Differentially expressed genes of GSE65194 dataset. Supplementary Table 6: Differentially expressed genes of TCGA BRCA dataset.
