# Supplementary figures and images for: LINC00467, Driven by Copy Number Amplification and DNA Demethylation, Is Associated with Oxidative Lipid Metabolism and Immune Infiltration in Breast Cancer
Source: Oxid Med Cell Longev. 2021 Dec 15;2021:4586319. doi: 10.1155/2021/4586319 (PMC8695024; doi:10.1155/2021/4586319)

Supplementary Figure 1

A

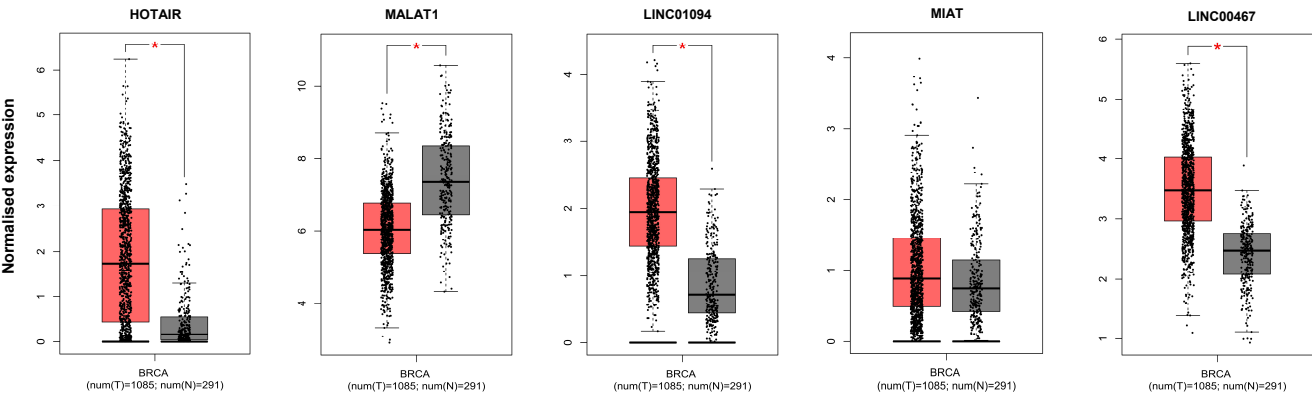

B

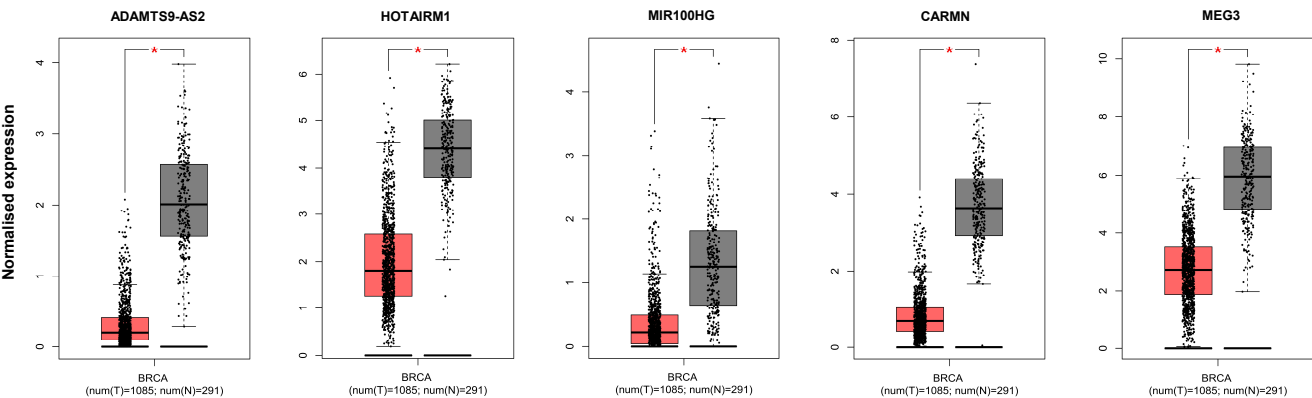

C

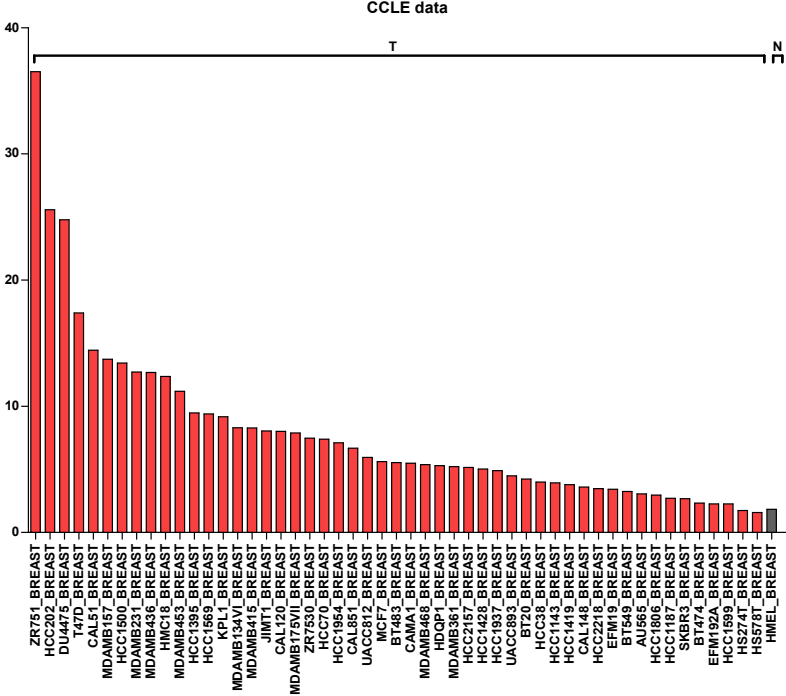

Supplement: Supplementary Materials — Supplementary Figure 1: Validation of differentially expressed lncRNAs in GEPIA BRCA dataset. (a, b) Differential expression was verified with GEPIA, with LINC00467 showing the most significant difference. (c) Expression of LINC00467 in BRCA cell line and normal mammary epithelial cell line of CCLE database. N: normal; T: tumor. Supplementary Figure 2: Expression of LINC00467 in CTCs of BRCA. LINC00467 expression in CTCs and BRCA in situ based on GSE41245. Supplementary Figure 3: TCGA Firehose Legacy dataset verifies the finding that LINC00467 had a high frequency of copy number amplification in BRCA. (a) LINC00467 has different frequencies of copy number amplification in different types of BRCA in Firehose Legacy dataset. (b) Relationship between LINC00467 expression and types of CNV. (c) The positive correlation between LINC00467 expression and its copy number level. (d) Patients with LINC00467 amplified had a worse overall survival. (e) The positive correlation between copy number amplification of LINC00467 and grades of BRCA. (f) Patients with LINC00467 amplified showed bigger lymph node examined number. (g) Patients with LINC00467 amplified showed more genomic changes. Supplementary Figure 4: WGCNA, enrichment, and correlation analysis. (a) Screening of the best soft thresholding with WGCNA. (b) Cell-specific and tissue-specific enrichment analysis of the genes at the red module where LINC00467 is located. (c) TF prediction of genes within the regulatory module. (d) Correlation analysis of LINC00467 and TGFB2 expression. Supplementary Table 1: Differentially expressed genes of GSE7904 dataset. Supplementary Table 2: Differentially expressed genes of GSE22820 dataset. Supplementary Table 3: Differentially expressed genes of GSE38959 dataset. Supplementary Table 4: Differentially expressed genes of GSE45827 dataset. Supplementary Table 5: Differentially expressed genes of GSE65194 dataset. Supplementary Table 6: Differentially expressed genes of TCGA BRCA dataset [file 4586319.f1.zip › 4586319.f1/Supplementary Figure 1.pdf]

# Supplementary Figure 2

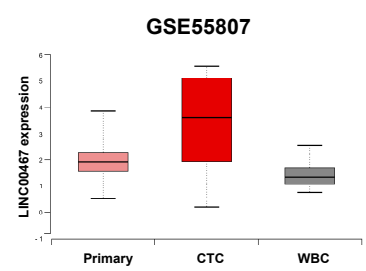

Supplement: Supplementary Materials — Supplementary Figure 1: Validation of differentially expressed lncRNAs in GEPIA BRCA dataset. (a, b) Differential expression was verified with GEPIA, with LINC00467 showing the most significant difference. (c) Expression of LINC00467 in BRCA cell line and normal mammary epithelial cell line of CCLE database. N: normal; T: tumor. Supplementary Figure 2: Expression of LINC00467 in CTCs of BRCA. LINC00467 expression in CTCs and BRCA in situ based on GSE41245. Supplementary Figure 3: TCGA Firehose Legacy dataset verifies the finding that LINC00467 had a high frequency of copy number amplification in BRCA. (a) LINC00467 has different frequencies of copy number amplification in different types of BRCA in Firehose Legacy dataset. (b) Relationship between LINC00467 expression and types of CNV. (c) The positive correlation between LINC00467 expression and its copy number level. (d) Patients with LINC00467 amplified had a worse overall survival. (e) The positive correlation between copy number amplification of LINC00467 and grades of BRCA. (f) Patients with LINC00467 amplified showed bigger lymph node examined number. (g) Patients with LINC00467 amplified showed more genomic changes. Supplementary Figure 4: WGCNA, enrichment, and correlation analysis. (a) Screening of the best soft thresholding with WGCNA. (b) Cell-specific and tissue-specific enrichment analysis of the genes at the red module where LINC00467 is located. (c) TF prediction of genes within the regulatory module. (d) Correlation analysis of LINC00467 and TGFB2 expression. Supplementary Table 1: Differentially expressed genes of GSE7904 dataset. Supplementary Table 2: Differentially expressed genes of GSE22820 dataset. Supplementary Table 3: Differentially expressed genes of GSE38959 dataset. Supplementary Table 4: Differentially expressed genes of GSE45827 dataset. Supplementary Table 5: Differentially expressed genes of GSE65194 dataset. Supplementary Table 6: Differentially expressed genes of TCGA BRCA dataset [file 4586319.f1.zip › 4586319.f1/Supplementary Figure 2.pdf]

Supplementary Figure 3

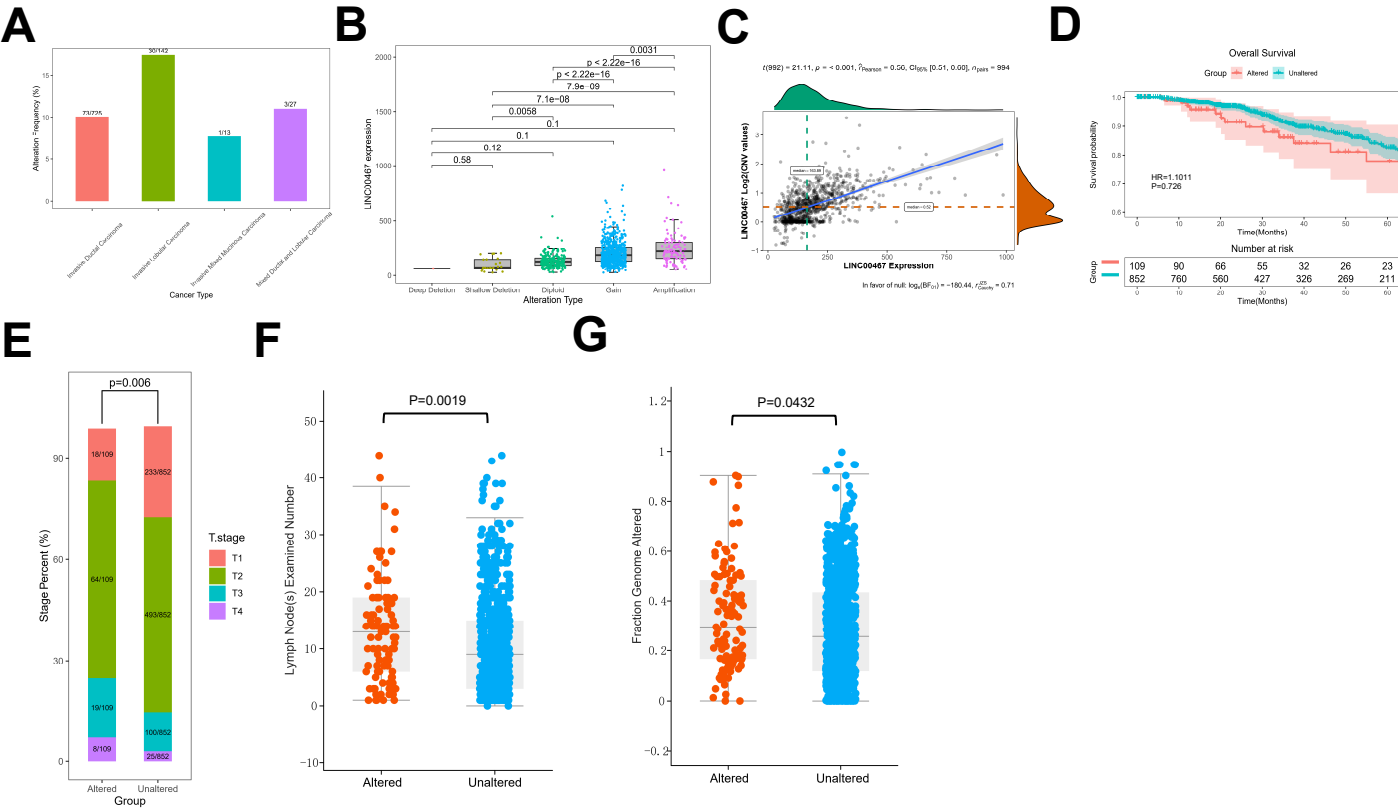

Supplement: Supplementary Materials — Supplementary Figure 1: Validation of differentially expressed lncRNAs in GEPIA BRCA dataset. (a, b) Differential expression was verified with GEPIA, with LINC00467 showing the most significant difference. (c) Expression of LINC00467 in BRCA cell line and normal mammary epithelial cell line of CCLE database. N: normal; T: tumor. Supplementary Figure 2: Expression of LINC00467 in CTCs of BRCA. LINC00467 expression in CTCs and BRCA in situ based on GSE41245. Supplementary Figure 3: TCGA Firehose Legacy dataset verifies the finding that LINC00467 had a high frequency of copy number amplification in BRCA. (a) LINC00467 has different frequencies of copy number amplification in different types of BRCA in Firehose Legacy dataset. (b) Relationship between LINC00467 expression and types of CNV. (c) The positive correlation between LINC00467 expression and its copy number level. (d) Patients with LINC00467 amplified had a worse overall survival. (e) The positive correlation between copy number amplification of LINC00467 and grades of BRCA. (f) Patients with LINC00467 amplified showed bigger lymph node examined number. (g) Patients with LINC00467 amplified showed more genomic changes. Supplementary Figure 4: WGCNA, enrichment, and correlation analysis. (a) Screening of the best soft thresholding with WGCNA. (b) Cell-specific and tissue-specific enrichment analysis of the genes at the red module where LINC00467 is located. (c) TF prediction of genes within the regulatory module. (d) Correlation analysis of LINC00467 and TGFB2 expression. Supplementary Table 1: Differentially expressed genes of GSE7904 dataset. Supplementary Table 2: Differentially expressed genes of GSE22820 dataset. Supplementary Table 3: Differentially expressed genes of GSE38959 dataset. Supplementary Table 4: Differentially expressed genes of GSE45827 dataset. Supplementary Table 5: Differentially expressed genes of GSE65194 dataset. Supplementary Table 6: Differentially expressed genes of TCGA BRCA dataset [file 4586319.f1.zip › 4586319.f1/Supplementary Figure 3.pdf]

# Supplementary Figure 4

A

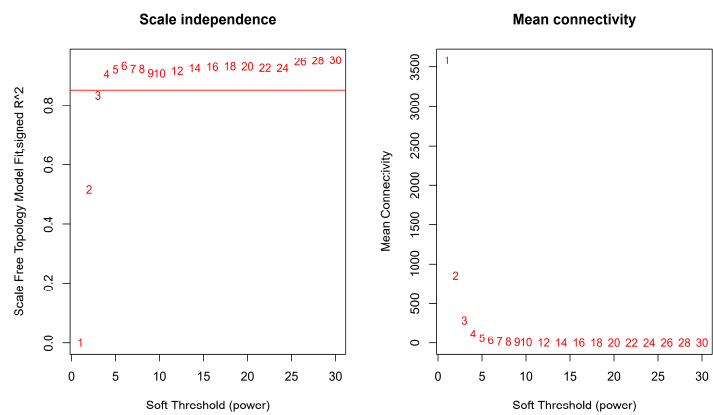

B

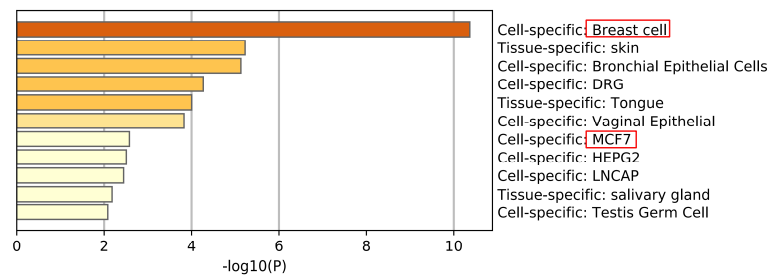

C

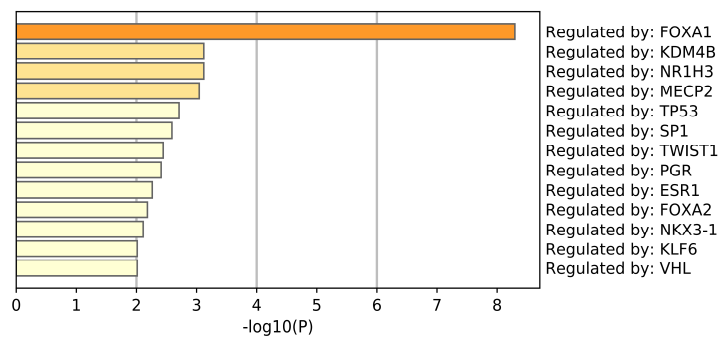

D

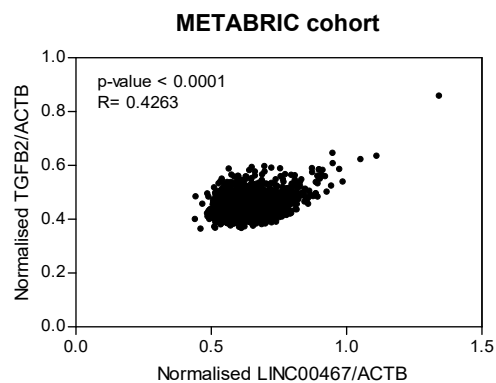

Supplement: Supplementary Materials — Supplementary Figure 1: Validation of differentially expressed lncRNAs in GEPIA BRCA dataset. (a, b) Differential expression was verified with GEPIA, with LINC00467 showing the most significant difference. (c) Expression of LINC00467 in BRCA cell line and normal mammary epithelial cell line of CCLE database. N: normal; T: tumor. Supplementary Figure 2: Expression of LINC00467 in CTCs of BRCA. LINC00467 expression in CTCs and BRCA in situ based on GSE41245. Supplementary Figure 3: TCGA Firehose Legacy dataset verifies the finding that LINC00467 had a high frequency of copy number amplification in BRCA. (a) LINC00467 has different frequencies of copy number amplification in different types of BRCA in Firehose Legacy dataset. (b) Relationship between LINC00467 expression and types of CNV. (c) The positive correlation between LINC00467 expression and its copy number level. (d) Patients with LINC00467 amplified had a worse overall survival. (e) The positive correlation between copy number amplification of LINC00467 and grades of BRCA. (f) Patients with LINC00467 amplified showed bigger lymph node examined number. (g) Patients with LINC00467 amplified showed more genomic changes. Supplementary Figure 4: WGCNA, enrichment, and correlation analysis. (a) Screening of the best soft thresholding with WGCNA. (b) Cell-specific and tissue-specific enrichment analysis of the genes at the red module where LINC00467 is located. (c) TF prediction of genes within the regulatory module. (d) Correlation analysis of LINC00467 and TGFB2 expression. Supplementary Table 1: Differentially expressed genes of GSE7904 dataset. Supplementary Table 2: Differentially expressed genes of GSE22820 dataset. Supplementary Table 3: Differentially expressed genes of GSE38959 dataset. Supplementary Table 4: Differentially expressed genes of GSE45827 dataset. Supplementary Table 5: Differentially expressed genes of GSE65194 dataset. Supplementary Table 6: Differentially expressed genes of TCGA BRCA dataset [file 4586319.f1.zip › 4586319.f1/Supplementary Figure 4.pdf]
